# From Bailout to Benchmark? Rethinking the Alfieri Procedure for Mitral Regurgitation in Barlow’s Disease

**DOI:** 10.3390/jcm15103818

**Published:** 2026-05-15

**Authors:** Karin Steiner, Bernhard Voss, Miriam Lang, Nikoleta Bozini, Spyridon Soulis, Martin Bichler, Maximilian-Niklas Bonk, Stephanie Voss, Keti Vitanova, Markus Krane, Konstantinos Sideris

**Affiliations:** 1 Department of Cardiovascular Surgery, TUM University Hospital German Heart Center, Technical University Munich, Lazarettstrasse 36, 80636 Munich, Germany; steinerk@dhm.mhn.de (K.S.); voss@dhm.mhn.de (B.V.); langmi@dhm.mhn.de (M.L.); bozini@dhm.mhn.de (N.B.); bichlerm@dhm.mhn.de (M.B.); bonk@dhm.mhn.de (M.-N.B.); vosss@dhm.mhn.de (S.V.); vitanova@dhm.mhn.de (K.V.); krane@dhm.mhn.de (M.K.); 2 Department of Cardiovascular Surgery, INSURE (Institute of Translational Cardiac Surgery), TUM University Hospital German Heart Center, TUM School of Medicine and Health, Technical University Munich, 80636 Munich, Germany; 3 DZHK (German Center for Cardiovascular Research) Partner Site Munich Heart Alliance, 80636 Munich, Germany

**Keywords:** Barlow’s disease, mitral regurgitation, mitral valve repair, Alfieri, edge–to–edge, Neochordae, annuloplasty

## Abstract

**Background:** Mitral regurgitation due to Barlow’s disease remains surgically demanding. Despite widespread experience, consensus is lacking on whether the Alfieri repair can serve as a deliberate and durable rather than a rescue strategy in this complex pathology. **Methods**: We retrospectively analyzed patients undergoing mitral valve repair due to severe mitral regurgitation resulting from Barlow’s disease using either the Alfieri or Neochordae repair techniques. Patients received a uniform semi–rigid annuloplasty ring, while leaflet resection and concomitant coronary or aortic procedures were excluded. **Results**: Baseline demographics and echocardiography were broadly comparable. Perioperative mortality was 0% in both cohorts, with similarly low rates of major complications. Aortic cross–clamp time was significantly shorter with Alfieri repair (*p* < 0.001). No relevant postoperative transmitral gradient or systolic anterior motion occurred. At a mean follow–up of 4.2 years, more–than–moderate MR was observed in one patient per group (Alfieri 2.4% vs. Neochordae 1.2%). At 10 years, the cumulative incidence of more–than–moderate mitral regurgitation and redo mitral surgery was similarly low between techniques (*p* = 0.810 and *p* = 0.460). Most patients were NYHA class I–II at last follow–up, demonstrating improved functional status. Echocardiography showed left ventricular reverse remodeling without intergroup differences. **Conclusions**: These data indicate that the Alfieri approach provides durable competence and hemodynamic safety comparable to the Neochordae technique while reducing cross–clamp time, supporting its use as a deliberate strategy rather than a bailout in anatomically suitable valves.

## 1. Introduction

Barlow’s disease represents a complex subtype of degenerative mitral valve (MV) disease and is a common cause of severe mitral regurgitation in young and middle–aged patients [1]. It is characterized by excessive leaflet tissue, bileaflet prolapse, elongated or ruptured chordae tendinae, and pronounced annular dilatation [1]. This marked anatomical heterogeneity poses significant technical challenges, reinforcing the need for repair strategies that are both reproducible and durable.

According to current guidelines, mitral valve repair is the preferred treatment for severe degenerative mitral regurgitation (MR) and is associated with improved survival, preservation of left ventricular (LV) function, as well as reduced valve–related complications [2,3]. As with other forms of degenerative mitral regurgitation, surgical management of Barlow valves relies on a combination of rigid or semi–rigid annuloplasty, implantation of artificial Neochordae, and leaflet resection [4]. While these techniques have demonstrated excellent long–term outcomes in experienced centers, they remain technically demanding, are associated with prolonged operative times, and variability of surgical results [5,6,7,8]. The edge–to–edge (E2E) repair described by O. Alfieri has been established as an alternative complementary surgical concept in MV repair [9]. By creating a functional double–orifice valve, this technique provides a simplified and durable approach to restoring leaflet coaptation, particularly in complex or multi–segment prolapse [4,6,10]. Further evidence argues against an inherent stenotic effect of the E2E construct and demonstrates the potential of Alfieri’s technique in determined Barlow cohorts [6,11,12,13]. Despite its widespread use in transcatheter mitral valve repair, the role of Alfieri’s concept in the surgical treatment of Barlow disease remains controversial and is supported by only limited comparative data. Consequently, it is still frequently deployed as a rescue strategy when conventional repairs become tenuous.

The present study addresses this gap by systematically comparing the deliberate use of the Alfieri technique head–to–head against Neochordae implantation for mitral valve repair in severe mitral regurgitation due to Barlow’s disease. We investigated whether the edge–to–edge approach provides comparable outcomes, including mid–term repair durability, hemodynamics, functional and perioperative results, emphasizing its role as a primary repair approach rather than a bailout maneuver.

## 2. Materials and Methods

### 2.1. Study Design

We conducted a single–center, retrospective cohort study of patients who underwent surgical repair for severe primary MR due to Barlow’s disease between 2007 and 2023. Data were obtained from the institutional database, operative reports, and referring cardiologists. To minimize confounding by device heterogeneity or repair method, we only included cases using a single semi–rigid annuloplasty ring and excluded leaflet resection techniques, resulting in a selected Barlow cohort. Patients with prior mitral surgery, concomitant coronary artery bypass grafting (CABG), aortic or aortic valve procedures, or active infective endocarditis were excluded. Patients were treated with ring annuloplasty combined either with Alfieri repair or implantation of Neochordae consisting of polytetrafluoroethylene.

### 2.2. Operative Techniques

Operative access was either through a minimally invasive cardiac lateral surgery technique or median sternotomy. The repair strategy followed a deliberate, preoperative plan based on lesion set and leaflet excess, at the surgeon’s discretion. Ring sizing followed inter–trigonal distance or inter–commissural distance, anterior leaflet height, and dynamic testing. Annuloplasty was exclusively performed with a Medtronic Simulus^®^ ring (Medtronic, Minneapolis, MN, USA), characterized by the smallest cranio–caudal to anterior–posterior ratio [14]. Intraoperative transesophageal echocardiography (TEE) guided repair adequacy. According to current guidelines, in cases with concomitant atrial fibrillation or at least moderate tricuspid valve regurgitation, cryoablation or tricuspid valve repair was performed with adapted ring sizing [2]. All operations used standard cardiopulmonary bypass with aortic cross–clamping and cold cardioplegia. Post–repair anticoagulation or antiplatelet therapy followed guideline–based care for degenerative MR repair [2].

### 2.3. Echocardiographic Examination

Transthoracic echocardiography (TTE) was performed preoperatively, at discharge, and during follow–up. TEE was performed during surgery, before and after MV repair. Barlow pathology was defined as proposed by Carpentier [15]. MR severity was graded by an integrated approach using qualitative, semi–quantitative, and quantitative parameters, based on contemporary guidelines [2]. Systolic anterior motion (SAM) was defined as leaflet–septal contact on echocardiography with LVOT flow acceleration and/or gradient. Transmitral gradients were measured by continuous–wave Doppler, and clinically relevant stenosis was prespecified as a mean gradient ≥ 5 mmHg or valve area ≤ 1.5 cm^2^. LV reverse remodeling was assessed by LV ejection fraction (LV EF) and LV end–diastolic diameters (LVEDd), with sex–specific cutoffs for enlargement and severe enlargement [16].

### 2.4. Outcomes

The primary endpoint was durability of surgical repair, defined as freedom from the composite of more–than–moderate MR or MV reoperation at follow–up. Further outcomes included mean transmitral gradient, occurrence of SAM, echocardiographic reverse remodeling over time, operative efficacy, and clinical status (NYHA class). Device success was reported according to the Mitral Valve Academic Research Consortium (MVARC) definition [17].

### 2.5. Statistical Analysis

Continuous variables were expressed as mean ± standard deviation, while categorical data were presented as frequencies and percentages. Comparisons between groups were performed using Welch’s t–test for continuous variables and the Chi–square test or Fisher’s exact test for categorical variables. Paired changes from baseline to follow–up were evaluated using the Wilcoxon signed–rank test. The composite endpoint was analyzed using risk ratios with 95% confidence intervals and Fisher’s exact test. Cumulative incidence curves were used to illustrate event occurrence over time, whereas between–group differences were formally assessed using log–rank testing and Cox proportional hazard models, reporting hazard ratios with 95% confidence intervals. The global significance level was chosen as *p* < 0.05. Analyses were conducted in Python (3.12) and in R (4.2).

## 3. Results

### 3.1. Study Cohort

**Figure 1:**
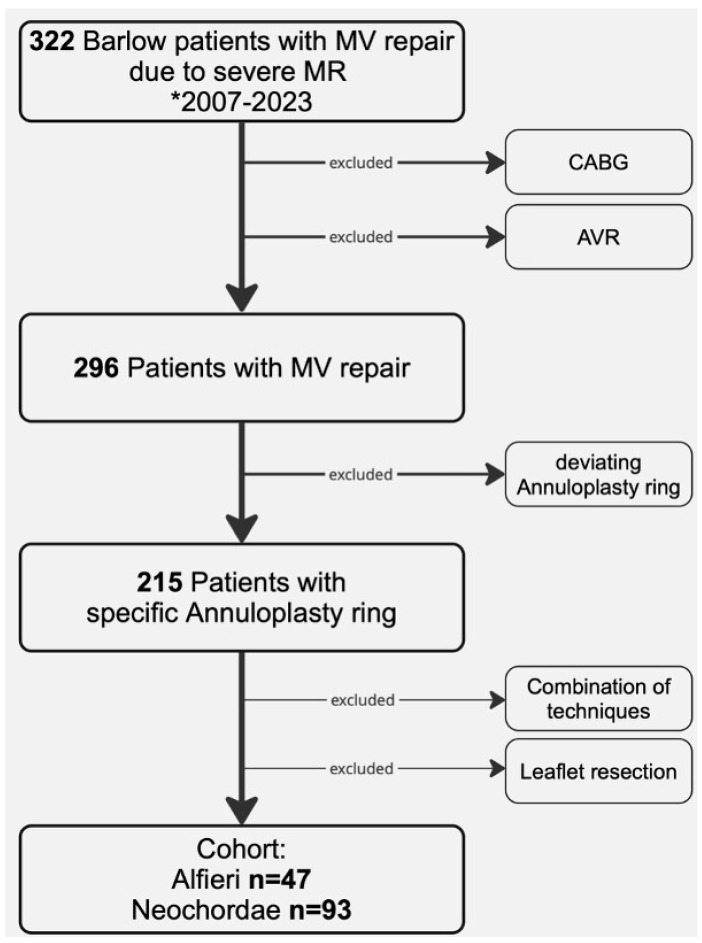
Patient selection. A total of 322 patients with Barlow’s disease underwent surgical mitral valve repair between 2007–2023 (* Inclusion period). Exclusion of CABG, concomitant aortic or aortic valve procedures (AVR), and deviating annuloplasty rings. Further exclusion of leaflet resection techniques or a combination of repair techniques resulted in an analytic cohort of 140. Repair by either the Alfieri (*n* = 47) or Neochordae (*n* = 93) technique.

From 322 patients with Barlow’s disease undergoing surgical mitral valve repair, cases with concomitant CABG, concomitant aortic valve repair or replacement (AVR), or deviating annuloplasty ring systems were excluded, yielding 215 patients treated with a uniform semi–rigid annuloplasty ring (Figure 1). After further excluding leaflet resections and combinations of different repair techniques, the analytic cohort comprised 140 patients treated with annuloplasty combined with either Alfieri suturing (*n* = 47) or implantation of Neochordae (*n* = 93).

### 3.2. Baseline Characteristics

The baseline demographic and clinical profiles were comparable between groups (Table 1). Patients were middle–aged (54.7 ± 9.0 vs. 51.9 ± 13.1 years, *p* = 0.141) and one–third were female (29.8% vs. 33.3%; *p* = 0.816). Functional status was similar, with most patients in NYHA class I or II (40.0% and 37.9%). Surgical risk was low in both cohorts (EuroSCORE II 1.3 ± 0.9 vs. 1.6 ± 1.6, *p* = 0.260). Pulmonary hypertension was more frequent in the Neochordae group without significance (17.0% vs. 28.0%; *p* = 0.224), COPD was uncommon (0% vs. 4.3%; *p* = 0.551), and chronic kidney disease was rare (2.1% vs. 2.2%, *p* = 1.00). Atrial fibrillation tended to be more prevalent in the Alfieri group (31.9% vs. 17.2%), without reaching significance (*p* = 0.077). Cardiac conduction disease and device therapy were infrequent and comparable (atrioventricular block 4.3% vs. 5.4%; pacemaker 2.1% vs. 3.2%; implantable cardioverter defibrillator 2.1% vs. 1.1%).

Baseline echocardiographic characteristics were largely comparable between cohorts (Table 2). LV EF was preserved in most patients (LV EF > 50%: 95.7% vs. 94.6%, *p* = 1.00), with similar proportions of mildly reduced (41–49%) and reduced (<40%) LV EF. Left atrial enlargement was common (89.4% vs. 87.1%, *p* = 0.910), and left ventricular end–diastolic diameter was enlarged in over half (63.8% vs. 58.1%, *p* = 0.635) of patients. Mild aortic regurgitation (AR) and greater–than–mild tricuspid regurgitation (TR) were uncommon and similar. Leaflet pathology differed; while mainly posterior mitral leaflet prolapse (PML) was more common in the Neochordae group (47.3% vs. 14.9%, *p* < 0.001), bileaflet prolapse predominated in the Alfieri group (85.1% vs. 47.3%, *p* < 0.001). All patients had severe mitral regurgitation by design. Barlow valve morphologic phenotypes and mitral annular geometry are further summarized in Table 2.

### 3.3. Operative and Perioperative Data

Rates of concomitant tricuspid valve repair (21.3% vs. 20.4%, *p* = 1.00) and cryoablation were comparable (23.4% vs. 9.7%, *p* = 0.053, Table 3). Operative times were significantly shorter with Alfieri (cross–clamp 75.7 ± 27.2 vs. 100.3 ± 34.9 min., *p* < 0.001; bypass 122.7 ± 39.4 vs. 143.7 ± 48.4 min., *p* < 0.006). Main surgical access was performed by median sternotomy (Alfieri 93.6% vs. Neochordae 90.3%). There was no perioperative mortality, one intraoperative stroke without residual deficit, and early reoperation for bleeding was rare and similar (4.3% in both), with no early thromboembolic events, renal replacement therapy, relevant pericardial effusion, or new pacemaker implantation. Discharge mean transmitral gradient was clinically negligibly higher after Alfieri (2.9 ± 1.3 vs. 2.0 ± 0.9 mmHg, *p* < 0.001), and no SAM was observed on intraoperative TEE or discharge TTE.

### 3.4. Follow–Up Data and Functional Status

Echocardiographic and clinical follow–up was high with 88.6% (*n* = 124), at a mean of 4.2 (±2.0) years (Table 4). Comorbidities and adverse events were infrequent and similar between groups, including atrial fibrillation (9.8% vs. 13.2%; *p* = 0.770), stroke (7.3% vs. 13.2%; *p* = 0.774), myocardial infarction (0% vs. 2.4%; *p* = 0.547), and endocarditis (0% vs. 1.2%; *p* = 1.00). Most patients were NYHA class I (82.9% vs. 86.7%; *p* = 0.885), with no patients in class IV, with significant within–group improvement from baseline (Wilcoxon *p* < 0.001 for both; Supplemental Figure S1). Echocardiographic follow–up showed mostly preserved LV EF > 50% (95.1% vs. 89.2%; *p* = 0.164), with no patients with LV EF < 40%. MR severity remained low and was similarly distributed between Alfieri and Neochordae repair techniques, as summarized in Table 4.

### 3.5. Mid-Term Valve Gradients

**Figure 2:**
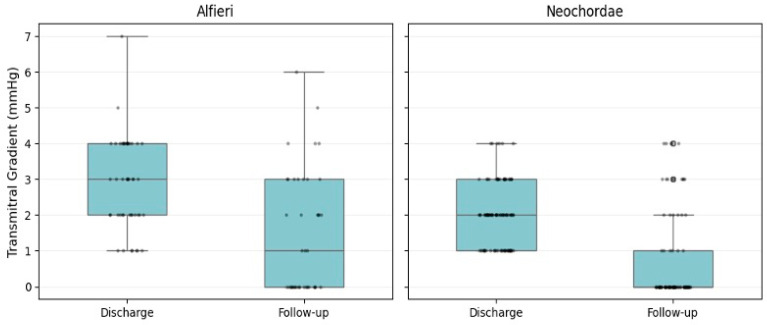
Evolution of transmitral pressure gradients after mitral valve repair at discharge and follow–up (mean 4.2 years) via box plot illustration, using either the Alfieri (**left**) or Neochordae (**right**) technique. The values presented are based on available data.

Transmitral pressure gradients remained low in both cohorts and decreased from discharge to follow–up (mean 4.2 years), with no case of clinically relevant late gradient (Figure 2) or SAM. The mean gradient at follow–up was 1.0 ± 1.5 mmHg overall, 1.5 ± 1.7 mmHg after Alfieri repair, and 0.8 ± 1.3 mmHg after Neochordae implantation (Table 4). While follow–up gradients were numerically higher in the Alfieri group, the absolute values were small and clinically negligible (*p* = 0.021; Table 4). Within–group comparisons showed significant improvement of mean transmitral gradient, Wilcoxon signed–rank test: Alfieri *p* < 0.001, Neochordae repair *p* < 0.001.

### 3.6. Reverse Remodeling and Systolic Function

**Figure 3:**
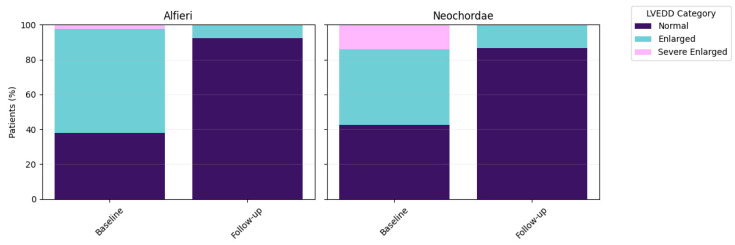
Left ventricular reverse remodeling after mitral valve repair at baseline and follow–up (mean 4.2 years). Stacked bar charts depict changes in left ventricular end–diastolic diameter (LVEDd) using either the Alfieri (**left**) or Neochordae (**right**) technique. LVEDd was categorized as normal, enlarged, or severely enlarged (LVEDD category). The percentages are based on available data.

Left ventricular reverse remodeling was observed in both cohorts from baseline to follow–up (mean 4.2 years; Figure 3). Within–group paired comparisons confirmed significant reductions in LVEDd (Wilcoxon signed–rank test: Alfieri *p* < 0.001, Neochordae *p* < 0.001).

**Figure 4:**
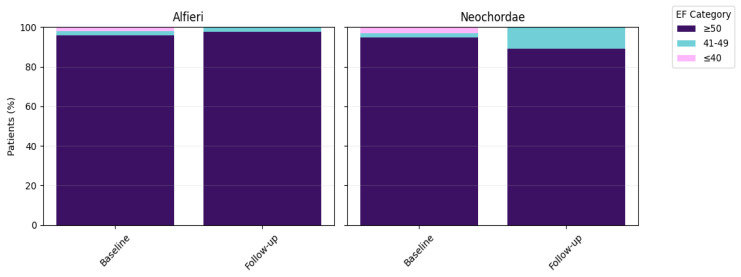
Evolution of left ventricular ejection fraction (LV EF) after mitral valve repair at baseline and follow–up (mean 4.2 years). Stacked bar charts illustrate changes in LV EF using either the Alfieri (**left**) or Neochordae (**right**) technique. LV EF was categorized (EF category) as preserved (≥50%), mildly reduced (41–49%), or moderately to severely reduced (≤40%). Percentages are based on available data.

Left ventricular systolic function remained stable from baseline to follow–up (mean 4.2 years) in both surgical cohorts (Figure 4). Most patients maintained preserved ejection fraction (≥50%) after repair with either the Alfieri edge–to–edge technique or Neochordae repair (Table 4; Figure 4). Within–group comparisons showed no significant change in LV EF over time (Wilcoxon signed–rank test: Alfieri *p* = 0.655, Neochordae replacement *p* = 0.523).

### 3.7. Repair Durability

**Figure 5:**
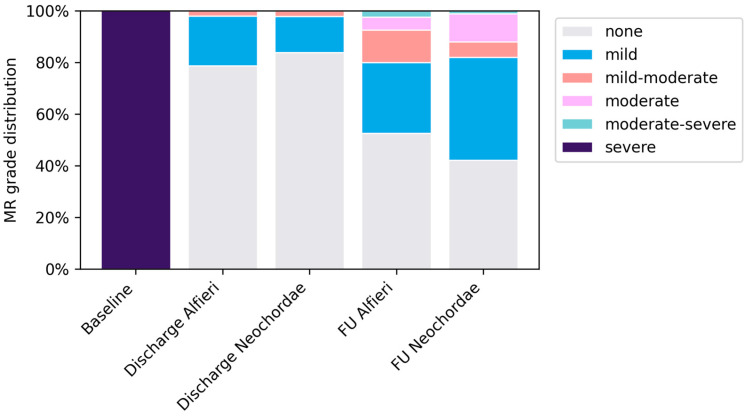
Evolution of mitral regurgitation severity in the Alfieri and Neochordae cohorts at baseline, hospital discharge, and follow–up (mean 4.2 years) after mitral valve repair. MR severity was graded as none, mild, mild–to–moderate, moderate, or moderate–to–severe. Values presented are based on available data.

At discharge, 97.9% of Alfieri and 98.9% of Neochordae patients had none or mild MR, with only one patient per group exhibiting mild–to–moderate MR (Figure 5). At a mean follow–up of 4.2 years, MR grades remained low, with more–than–moderate MR in one patient per group (Alfieri 2.4% vs. Neochordae 1.2%, *p* = 0.546; Table 4, Figure 5). Reoperation was infrequent (Alfieri 2.6% vs. Neochordae 3.8%, *p* = 1.00; Table 4), and MVARC device success was high at 30 days (Alfieri 95.74% vs. Neochordae 95.69%) and similar at follow–up (Alfieri 82.98% vs. Neochordae 83.87%). The composite endpoint (reoperation or more–than–moderate MR at follow–up) occurred infrequently and equally (two of 47 [4.3%] vs. four of 93 [4.3%]; risk ratio 0.99, 95% confidence interval (CI) 0.19–5.21; Fisher’s exact *p* = 1.00), without a statistically significant association between surgical method and the composite outcome.

**Figure 6:**
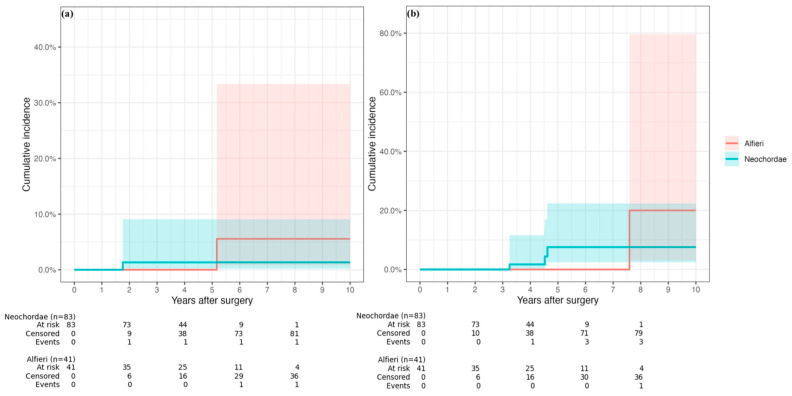
Cumulative incidence curves compare the 10–year risk of more–than–moderate mitral regurgitation (**a**) and reoperation (**b**) after surgical mitral valve repair in patients treated with Neochordae or Alfieri techniques. Shaded bands indicate 95% confidence intervals.

Ten–year cumulative incidence of recurrent more–than–moderate MR was low and comparable between techniques (log–rank *p* = 0.810), with no difference on Cox analysis (hazard ratio (HR) 1.42, 95% CI 0.08–24.11; *p* = 0.810), and 5–year risk of 0% after Alfieri and 1.3% after Neochordae implantation (Figure 6). Likewise, redo mitral valve surgery was infrequent and comparable (log–rank *p* = 0.460; Cox HR 0.36, 95% CI 0.03–3.82; *p* = 0.394), and 5–year risk was 0% in the Alfieri group and 7.6% in the Neochordae group, with wide confidence intervals reflecting low event rates (Figure 6).

## 4. Discussion

### 4.1. Context

Barlow’s disease has traditionally favored intricate, multisegmental repairs with substantial technical variability [5,6,7,8,18]. The Alfieri concept offers a simplified alternative to restore coaptation, yet concerns regarding restricted leaflet motion and functional stenosis in redundant valves have contributed to persistent skepticism and the tendency to deploy it as a rescue maneuver [4,6,10,12,19]. Despite broad adoption of edge–to–edge repair in transcatheter therapy, direct comparative surgical head–to–head data in Barlow disease, particularly under standardized annuloplasty, remain scarce [6,11,12,13]. Against this background, we examined whether deliberate E2E repair with a uniform semi–rigid annuloplasty ring can provide durable repair and preserved hemodynamics in comparison with Neochordae implantation. Our results support this premise, while underscoring that technique selection remains anatomy– and surgeon–dependent.

### 4.2. Principal Findings

Deliberate Alfieri repair achieved excellent early– and mid–term freedom from recurrent more–than–moderate MR or reoperation, similar to Neochordae implantation, while significantly shortening cross–clamp and cardiopulmonary bypass times. Transmitral gradients remained low throughout follow–up, and no SAM occurred, indicating preserved valve hemodynamics and addressing the concern that operative efficiency with E2E might come at the cost of clinically relevant trade–offs in redundant Barlow valves [12]. The central image that emerges is a pragmatic one: under uniform ring annuloplasty in a pure Barlow cohort, Alfieri repair can function as an efficient, reproducible primary strategy.

### 4.3. Baseline Phenotype

Baseline lesion phenotype differed between cohorts, consistent with potential confounding by indication: bileaflet prolapse predominated in the Alfieri group, whereas more pronounced posterior prolapse was more frequent in the Neochordae group. Accordingly, these comparative findings should be interpreted with caution. Nonetheless, both cohorts represented the same underlying disease entity, Barlow’s disease, with comparable annular geometry between groups and commissural–to–anteroposterior ratio near one, consistent with typical Barlow annular remodeling [20]. This supports that the comparison is anchored within a uniform degenerative phenotype rather than mixing different subtypes. Additionally, the lesion imbalance towards a more complex prolapse would be expected to bias against Alfieri outcomes, yet durability was comparable, supporting E2E efficacy also in complex anatomy. Importantly, these findings do not imply universal superiority of Alfieri, but comparable results in anatomically selected valves at the surgeon’s discretion [4,6,9,10,19].

### 4.4. Hemodynamic Considerations

Concerns that E2E repair may predispose to functional stenosis or restricted leaflet motion are particularly relevant in tissue–redundant Barlow valves [4,6,10,19]. In our cohort, no patient developed a clinically relevant late transmitral gradient at follow–up, and SAM was not observed. Although gradients were numerically higher after Alfieri, absolute values were small and clinically negligible, supporting preserved hemodynamics without iatrogenic stenosis. These observations are consistent with reports that E2E hemodynamic safety depends on concomitant annuloplasty strategy and appropriate sizing [6,10,11,12,13,19].

### 4.5. Durability, Remodeling, and Functional Recovery

Both strategies achieved sustained MR reduction at discharge and mid–term follow–up, with rare reoperation or more–than–moderate recurrent MR, indicating durable repair in Barlow disease. However, low event rates, wide confidence intervals, and sparse late follow–up limit power and warrant cautious interpretation of comparative long–term durability. Overall, these results translated into significant clinical recovery: most patients were NYHA I or II at follow–up. Both cohorts also showed significant LV reverse remodeling with reduced ventricular dilation and stable, preserved ejection fraction, consistent with restoration of LV geometry after effective MR elimination. These results align with prior comparative studies showing similar MR recurrence across E2E and conventional repair strategies, and with long–term series supporting durable E2E performance in selected degenerative cohorts [6,10,11,12,13,19].

### 4.6. Operative Efficiency and Clinical Implications

The Alfieri strategy yielded significantly shorter ischemic and extracorporeal support times, which may be advantageous when operative time is critical. Greater procedural efficiency may also improve reproducibility and reduce inter–surgeon variability in complex Barlow repair [5,6,7,8]. This aligns with current trends toward standardized repairs without compromising durability [2,4]. While our results support deliberate E2E, the repair strategy should remain individualized, integrating valve anatomy, annular geometry, and institutional expertise.

### 4.7. Limitations

This analysis is limited by its non–randomized, retrospective, single–center design. Despite broadly comparable baseline characteristics and a standardized annuloplasty system, residual surgeon selection bias cannot be excluded, particularly given differences in lesion localization. The follow–up echocardiographic assessment reflects mid–term rather than long–term durability and was not exclusively performed by a core laboratory. Event rates for reoperation and more–than–moderate MR were low, limiting statistical power for rare endpoints, while sparse late follow–up limits interpretation of long–term cumulative incidence estimates. Finally, findings may not be generalizable to centers employing different annuloplasty systems or operative philosophies. Prospective multicenter studies with standardized echocardiography protocols and longer follow–up are warranted and would be valuable to confirm the results and define patient–specific predictors of success.

## 5. Conclusions

Because the Alfieri approach is often used as a rescue in difficult anatomy, its outcomes are confounded. Our standardized cohort reduces this bias, suggesting that the Alfieri technique can serve as a deliberate and feasible strategy in patients with severe mitral regurgitation undergoing repair in selected Barlow valves. The findings should be interpreted with caution, given the residual selection bias, low event rates, and limited late follow–up, but support Alfieri as a surgeon–directed option in anatomically suitable valves, rather than as a bailout maneuver. Both Neochordae and Alfieri techniques yielded excellent outcomes, while clinical improvement paralleled reverse left ventricular remodeling and sustained MR reduction over follow–up. Notably, the Alfieri approach, while procedurally simpler, achieved greater operative efficiency without compromising mid–term durability. Therefore, in experienced centers, standardized E2E with ring annuloplasty offers a reproducible pathway to competence and streamlined operative workflow, broadening the armamentarium for degenerative MR. Confirmation in multicenter prospective cohorts with extended follow–up is needed.

## Figures and Tables

**Table 1:** Clinical baseline characteristics. NYHA = New York Heart Association, COPD = chronic obstructive pulmonary disease, AV–Block = atrioventricular block, ICD = implantable cardioverter defibrillator. Values are presented as *n* (%) or mean ± SD. Percentages are based on available data, missing values are excluded from the denominator.

	Overall *n* = 140	Alfieri *n* = 47	Neochordae *n* = 93	*p*–Value
Female gender, *n* (%)	45 (32.1)	14 (29.8)	31 (33.3)	0.815
Age, years (mean ± SD)	52.9 (±11.9)	54.7 (±9.0)	51.9 (±13.1)	0.141
NYHA class I, *n* (%)	56 (40.0)	17 (36.2)	39 (41.9)	0.635
NYHA class II, *n* (%)	53 (37.9)	20 (42.6)	33 (35.5)	0.529
NYHA class III, *n* (%)	30 (21.4)	11 (23.4)	19 (20.4)	0.852
NYHA class IV, *n* (%)	3 (2.1)	1 (2.1)	2 (2.2)	1.000
Euroscore II (mean ± SD)	1.5 (±1.4)	1.3 (±0.9)	1.6 (±1.6)	0.259
Pulmonal Hypertension, *n* (%)	34 (24.3)	8 (17.0)	26 (28.0)	0.224
COPD, *n* (%)	3 (3.0)	0 (0.0)	3 (4.3)	0.551
Atrial fibrillation, *n* (%)	31 (22.1)	15 (31.9)	16 (17.2)	0.076
AV–Block, *n* (%)	7 (5.0)	2 (4.3)	5 (5.4)	1.000
Pacemaker, *n* (%)	4 (2.9)	1 (2.1)	3 (3.2)	1.000
ICD, *n* (%)	2 (1.4)	1 (2.1)	1 (1.1)	1.000
Chronic kidney disease, *n* (%)	3 (2.2)	1 (2.2)	2 (2.2)	1.000
Dialysis, *n* (%)	0 (0.0)	0 (0.0)	0 (0.0)	

**Table 2:** Baseline echocardiographic data. LV EF = left ventricular ejection fraction, LAd = left atrial diameter, LVEDd = left ventricular end–diastolic diameter, AML = anterior mitral leaflet, PML = posterior mitral leaflet, AR = aortic regurgitation, TR = tricuspid regurgitation, MR = mitral regurgitation, CC = commissural, AP = anterior–posterior, MV–ratio = mitral valve ratio. Values are presented as *n* (%) or mean ± SD. Percentages are based on available data; missing values are excluded from the denominator.

	Overall *n* = 140	Alfieri *n* = 47	Neochordae *n* = 93	*p*–Value
LV EF > 50, *n* (%)	133 (95.0)	45 (95.7)	88 (94.6)	1.000
LV EF 41–49, *n* (%)	3 (2.1)	1 (2.1)	2 (2.2)	1.000
LV EF < 40, *n* (%)	4 (2.9)	1 (2.1)	3 (3.2)	1.000
Enlarged LAd, *n* (%)	123 (87.9)	42 (89.4)	81 (87.1)	0.909
Enlarged LVEDd, *n* (%)	84 (60)	30 (63.8)	54 (58.1)	0.635
Mainly AML prolapse, *n* (%)	5 (3.6)	0 (0)	5 (5.4)	0.167
Mainly PML prolapse, *n* (%)	51 (36.4)	7 (14.9)	44 (47.3)	<0.001
Bileaflet prolapse, *n* (%)	84 (60.0)	40 (85.1)	44 (47.3)	<0.001
Mild AR, *n* (%)	25 (17.9)	7 (14.9)	18 (19.4)	0.677
TR > mild, *n* (%)	17 (12.1)	6 (12.8)	11 (11.8)	0.463
Severe MR, *n* (%)	140 (100)	47 (100)	93 (100)	
CC diameter, mm (mean ± SD)	35.9 (±19.7)	34.8 (±22.1)	36.5 (±18.6)	0.652
AP diameter, mm (mean ± SD)	31.0 (±17.5)	29.7 (±1 8.9)	31.7 (±16.8)	0.533
MV–ratio (CC/AP) (mean ± SD)	1.2 (±0.1)	1.2 (±0.1)	1.1 (±0.1)	0.114

**Table 3:** Perioperative data. TVR = tricuspid valve repair, MV = mitral valve. Values are presented as *n* (%) or mean ± SD. Percentages are based on available data; missing values are excluded from the denominator.

	Overall *n* = 140	Alfieri *n* = 47	Neochordae *n* = 93	*p*–Value
Bypass time, minutes (mean ± SD)	136.6 (±46.5)	122.7 (± 39.4)	143.7 (±48.4)	0.006
Cross–clamp time, minutes (mean ± SD)	92.0 (±34.5)	75.7 (±27.2)	100.3 (±34.9)	<0.001
Ischemia time, minutes (mean ± SD)	89.7 (±32.8)	74.5 (±26.4)	97.3 (±33.1)	<0.001
Reperfusion time, minutes (mean ± SD)	29.2 (±13.1)	31.3 (±13.8)	28.1 (±12.7)	0.180
Median sternotomy, *n* (%)	128 (91.4)	44 (93.6)	84 (90.3)	0.751
Minimally invasive access, *n* (%)	12 (8.6)	3 (6.4)	9 (9.7)	0.751
Ring size, mm (mean ± SD)	37.8 (±2.5)	38.6 (±2.2)	37.5 (±2.6)	0.010
Concomitant TVR, *n* (%)	29 (20.0)	10 (21.3)	19 (20.4)	1.000
Concomitant Cryoablation, *n* (%)	20 (13.8)	11 (23.4)	9 (9.7)	0.053
Reoperation for bleeding, *n* (%)	6 (4.3)	2 (4.3)	4 (4.3)	1.000
MV gradient, mmHg (mean ± SD)	2.3 (±1.1)	2.9 (±1.3)	2.0 (±0.9)	<0.001

**Table 4:** Follow–up characteristics. NYHA = New York Heart Association, MV = mitral valve, TTE = transthoracic echocardiography, LV EF = left ventricular ejection fraction, LVEDd = left ventricular end–diastolic diameter, MR = mitral regurgitation, TR = tricuspid regurgitation. Values are presented as *n* (%) or mean ± SD.

	Overall *n* = 124	Alfieri *n* = 41	Neochordae *n* = 83	*p*-Value
NYHA class I, *n* (%)	106 (85.4)	34 (82.9)	72 (86.7)	0.885
NYHA class II, *n* (%)	15 (12.1)	5 (12.2)	10 (12.0)	1.000
NYHA class III, *n* (%)	1 (0.8)	1 (2.4)	0 (0.0)	0.331
Atrial fibrillation, *n* (%)	15 (12.1)	4 (9.8)	11 (13.2)	0.770
Stroke, *n* (%)	14 (11.3)	3 (7.3)	11 (13.2)	0.774
Myocardial infarction, *n* (%)	2 (1.6)	0 (0.0)	2 (2.4)	0.547
Infectious endocarditis, *n* (%)	1 (0.8)	0 (0.0)	1 (1.2)	1.000
Reoperation MV, *n* (%)	4 (3.2)	1 (2.4)	3 (3.6)	1.000
Follow-up TTE, years (mean ± SD)	4.2 (±2.0)	4.6 (±2.4)	4.0 (±1.7)	0.159
LV EF > 50, *n* (%)	113 (91.1)	39 (95.1)	74 (89.2)	0.164
LV EF 41–49, *n* (%)	10 (8.1)	1 (2.4)	9 (10.8)	0.164
Enlarged LVEDd, *n* (%)	14 (11.3)	3 (7.3)	11 (13.3)	0.544
No MR, *n* (%)	36 (29.0)	13 (31.7)	23 (27.7)	0.737
None–mild MR, *n* (%)	20 (16.1)	8 (19.5)	12 (14.5)	0.603
Mild MR, *n* (%)	44 (35.5)	11 (26.8)	33 (39.8)	0.259
Mild–moderate MR, *n* (%)	10 (8.1)	5 (12.2)	5 (6.0)	0.379
Moderate MR, *n* (%)	11 (8.9)	2 (4.8)	9 (10.8)	0.500
Moderate–severe MR, *n* (%)	2 (1.6)	1 (2.4)	1 (1.2)	0.546
MV gradient, mmHg (mean ± SD)	1.0 (±1.5)	1.5 (±1.7)	0.8 (±1.3)	0.021
TR > mild, *n* (%)	1 (0.8)	0 (0.0)	1 (1.2)	1.000

## Data Availability

Due to privacy restrictions, raw data cannot be provided as supplementary material, but de–identified data are available from the corresponding author upon reasonable request.

## Supplementary Materials

The following supporting information can be downloaded at: https://www.mdpi.com/article/10.3390/jcm15103818/s1, Supplemental Figure S1. Evolution of Functional Status. Stacked bar charts depict changes in New York Heart Association (NYHA) functional class from baseline to follow-up (mean 4.2 years) in patients undergoing mitral valve repair using either the Alfieri edge-to-edge technique (left) or Neochordae repair (right). Percentages are based on available data.

## Abbreviations

The following abbreviations are used in this manuscript:

MV	Mitral valve
MR	Mitral regurgitation
E2E	Edge–to–edge
CABG	Coronary artery bypass grafting
TEE	Transesophageal echocardiography
TTE	Transthoracic echocardiography
SAM	Systolic anterior motion
LV EF	Left ventricular ejection fraction
LVEDd	Left ventricular end–diastolic diameter
NYHA	New York Heart Association
MVARC	Mitral Valve Academic Research Consortium
AVR	Aortic valve replacement
COPD	Chronic obstructive pulmonary disease
AV–Block	Atrioventricular block
ICD	Implantable cardioverter defibrillator
AR	Aortic regurgitation
TR	Tricuspid valve regurgitation
AML	Anterior mitral leaflet
PML	Posterior mitral leaflet
CC	Commissural
AP	Anterior–posterior
TVR	Tricuspid valve repair
CI	Confidence interval
HR	Hazard ratio
