# Supplementary material for: From Bailout to Benchmark? Rethinking the Alfieri Procedure for Mitral Regurgitation in Barlow’s Disease
Source: J Clin Med. 2026 May 15;15(10):3818. doi: 10.3390/jcm15103818 (PMC13207406; doi:10.3390/jcm15103818)
Supplement: Supplementary file 1 [file jcm-15-03818-s001.zip › jcm-4248515-supplementary.pdf]

Supplemental Material

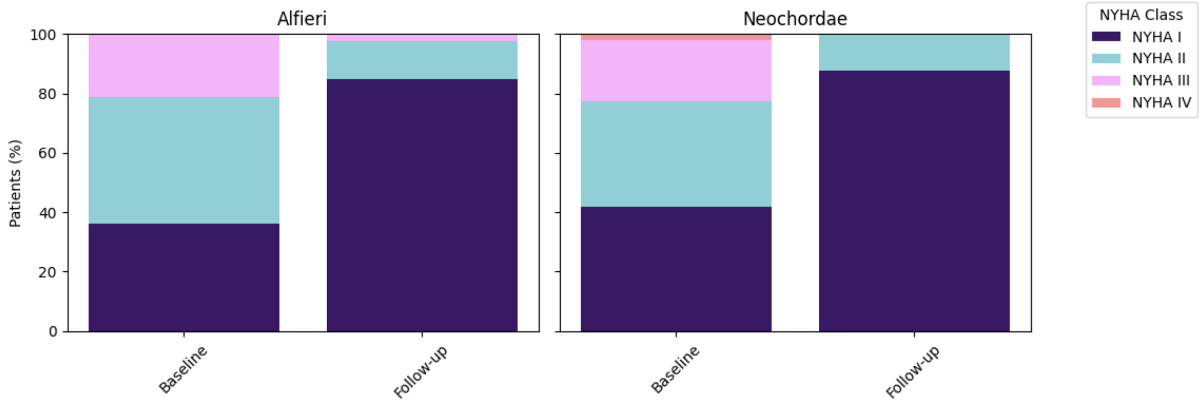

Supplemental Figure S1. Evolution of Functional Status. Stacked bar charts depict changes in New York Heart Association (NYHA) functional class from baseline to follow-up (mean 4.2 years) in patients undergoing mitral valve repair using either the Alfieri edge-to-edge technique (left) or Neochordae repair (right). Percentages are based on available data.
